# The association between acute transverse myelitis and COVID‐19 vaccination in Korea: Self‐controlled case series study

**DOI:** 10.1111/ene.70020

**Published:** 2024-12-30

**Authors:** Eunsun Lim, Yoo Hwan Kim, Na‐Young Jeong, Su‐Hyun Kim, Heehyun Won, Jong‐Seok Bae, Nam‐Kyong Choi, COVID‐19 Vaccine Safety Committee (CoVaSC).

**Affiliations:** ^1^ Department of Health Convergence, College of Science and Industry Convergence Ewha Womans University Seoul Republic of Korea; ^2^ Department of Neurology, Hallym University Sacred Heart Hospital, College of Medicine Hallym Universit Anyang Republic of Korea; ^3^ Department of Neurology Research Institute and Hospital of National Cancer Center Goyang Republic of Korea; ^4^ Department of Neurology, Kangdong Sacred Heart Hospital, College of Medicine Hallym University Seoul Republic of Korea; ^5^ Graduate School of Industrial Pharmaceutical Science, College of Pharmacy Ewha Womans University Seoul Republic of Korea

**Keywords:** adverse events, COVID‐19, self‐controlled case series, transverse myelitis, vaccination

## Abstract

**Background:**

Acute transverse myelitis (ATM) has been reported as a potential association between COVID‐19 vaccination. In this study, we aimed to investigate the association between the COVID‐19 vaccination and ATM.

**Methods:**

A self‐controlled case series study was performed using a large database that combine the COVID‐19 vaccine registry and the national claims database. The COVID‐19 vaccination data included information on individuals aged 18 and above who received COVID‐19 vaccination from February 26, 2021, to August 31, 2022. The claims database covered the entire Korean population for the period between January 1, 2002 to August 31, 2022. Patients who develop ATM within 1–42 days following COVID‐19 vaccination were included. The observation period was 270 days after the first dose of the COVID‐19 vaccine. The incidence rate ratio (IRR) and 95% confidence interval (CI) were estimated using a conditional Poisson regression model.

**Results:**

A total of 159 ATM patients were included. Among them, 82 (51.6%) were male, and mean age was 55.4 (±17.4) years. The IRR was 2.41 (95% CI: 1.76–3.30) for the ATM risk within 1–42 days after COVID‐19 vaccination. The IRR by vaccine product was 3.31 (95% CI: 1.81–6.05) for ChAdOx1‐S; 1.99 (95% CI: 1.30–3.03) for BNT162b2; 2.57 (95% CI: 1.14–5.97) for mRNA‐1273; and 3.33 (95% CI: 0.30–36.44) for Ad26.COV2.S.

**Conclusion:**

These findings indicated an increased risk of ATM following COVID‐19 vaccination within 42 days. An association with the risk of ATM was found both for viral vector and mRNA vaccines.

## INTRODUCTION

The rapid development of COVID‐19 vaccines at an unprecedented pace has been instrumental in the global effort to curb the pandemic. Several COVID‐19 vaccines received emergency use authorization within a year of the outbreak of SARS‐CoV‐2 virus [1, 2, 3]. These vaccines have been proven effective in preventing COVID‐19 infections and complications [4]. However, like any medical intervention, vaccines can carry potential risks and the occurrence of AEs needs to be thoroughly evaluated to ensure safety [3].

Acute transverse myelitis (ATM) is a rare neurological disorder characterized by the inflammation of the spinal cord, which can lead to sensory and motor deficits, paralysis, and severe disability [5]. The global incidence of ATM is estimated to be one to eight cases per million people per year. The causes of ATM are uncertain; however, it is known to be associated with autoimmune nature triggered by various environmental factors, including vaccination [6].

Since the beginning of the SARS‐CoV‐2 vaccination, a few cases of transverse myelitis (TM) following vaccination have been reported. Based on case reports and spontaneous reporting systems from various countries such as the UK, Denmark, and Taiwan, several studies have indicated a small number of cases of ATM following COVID‐19 vaccination [4, 7, 8, 9, 10, 11, 12]. The occurrence of ATM cases led to a temporary suspension of the clinical trials for ChAdOx1 nCov‐19 in Brazil, South Africa, and the UK [11]. A study using the World Health Organization (WHO) VigiBase found a potential association between both mRNA‐based and vector‐based vaccines and ATM [13]. The European Medicines Agency recommended the inclusion of TM as a potential side effect of Ad26COV2.S in the Summary of Product Characteristics, following a review of the reported cases [14]. While self‐reporting and case reports are effective in detecting potential safety alerts, they are not suited for estimating the intensity of possible associations. Most previous studies identified ATM cases following COVID‐19 vaccination based on either case reports or case series. Therefore, population‐based research is required to generate epidemiological evidence and assess the association between the COVID‐19 vaccine and TM.

The objective of this study was to investigate the association between the COVID‐19 vaccination and ATM, employing a self‐controlled case series (SCCS) design using a nationwide database.

## METHODS

### Data source

We used a large database that combined the COVID‐19 vaccine registry and the national claims database. The COVID‐19 vaccine registry provided by the Korea Disease Control and Prevention Agency (KDCA) contains vaccination status, vaccine brand, dose of vaccination, and vaccination date for an estimated 42 million vaccine recipients from February 26, 2021, to August 31, 2022. The National Health Insurance (NHI) claims database offered by the National Health Insurance Service (NHIS) includes demographics, diagnosis, and treatment data for approximately 51 million Korean population, covering a timeline from January 1, 2002, to August 31, 2022. This database features information such as diagnosis codes (i.e., International Statistical Classification of Disease and Related Health Problems, Tenth Version [ICD‐10 codes]), procedure and operation codes, and dates of medical visits.

The NHI claims database in Korea, maintained by the Health Insurance Review & Assessment Service (HIRA), contains comprehensive data on demographics, diagnoses, treatments, and medical visits [15]. As part of Korea's universal health coverage system, nearly all residents are insured under the NHIS, ensuring extensive national coverage. The term “claims” refers to the administrative records generated by healthcare providers when submitting requests for reimbursement to the NHIS for services provided, evaluated by HIRA to ensure they meet established healthcare standards [16]. The NHIS has integrated these databases using resident registration numbers, enhancing data integrity and privacy by providing data in an anonymized format for research purposes. Our access to this de‐identified data was exclusively facilitated within the dedicated data analysis center of the NHIS, ensuring compliance with all relevant privacy guidelines and regulations [17].

### Study design and population

We used a SCCS design to evaluate the safety of COVID‐19 vaccines and the risk of ATM. The SCCS is a case‐only design used to mitigate the impact of time‐varying confounding by comparing the incidence of outcomes during various periods for the same individual, both the exposed and the unexposed periods [18]. The SCCS design relies on critical preconditions [19, 20]. First, the outcome must be recurrent and independent, or uncommon. This design is suitable for studying rare outcomes like ATM, particularly in situations where there is an insufficient number of non‐exposure controls, as is often the case in mass vaccination situations. Second, the likelihood of exposure must remain unaffected by the occurrence of the events. We excluded the TM cases in the pre‐vaccination period before the first COVID‐19 vaccination because TM could decrease the probability of the vaccination. Third, the incidence of an event should not precipitate the premature cessation of the observation period due to mortality. This assumption was satisfied since TM is rarely associated with increased mortality.

The study population included individuals aged 18 years or older who received the first COVID‐19 vaccine dose and patients newly diagnosed with ATM. We included individuals who received the first COVID‐19 vaccination from February 26, 2021, to December 4, 2021, to ensure an adequate follow‐up period. The COVID‐19 vaccine included monovalent BNT162b2 (Pfizer‐BioNTech), mRNA‐1273 (Moderna), ChAdOx1‐S (AstraZeneca), Ad26.COV2.S (Johnson & Johnson), and NVX‐CoV2373 (Novavax), which were authorized by the Ministry of Food and Drug Safety of the Republic of Korea during the study period. Individuals were excluded if they had incomplete medical or vaccination records, or if they had received their vaccination outside of the country.

Patients diagnosed with ATM within the observation period (i.e., 1–270 days after the first dose) were identified using the ICD‐10 code G37.3 (ATM in demyelinating disease of central nervous system) and procedure codes for ATM‐related procedures, such as cerebral spinal fluid (CSF) analysis or magnetic resonance imaging (MRI) of the spinal cord, within a same inpatient episode (Table S1). We excluded individuals with a history of ATM, neuromyelitis optica (NMO), multiple sclerosis, or optic neuritis from January 1, 2002, to the date of ATM diagnosis. People diagnosed with other ATM‐related diseases (e.g., acute disseminated encephalomyelitis [ADEM], encephalitis, encephalomyelitis) within 365 days prior to the diagnosis of ATM were excluded, to focus on newly diagnosed ATM cases following COVID‐19 vaccination (Table S1). We also excluded those who were diagnosed with COVID‐19 within 28 days prior to their ATM diagnosis to minimize confounding effects and better isolate the incidence of ATM possibly triggered by the vaccine rather than direct viral effects. This exclusion criterion aligns with the understanding that neurological manifestations, such as ATM, can occur due to the immune response to COVID‐19 typically manifesting 10 days to 6 weeks post‐infection [21].

**FIGURE 1:**
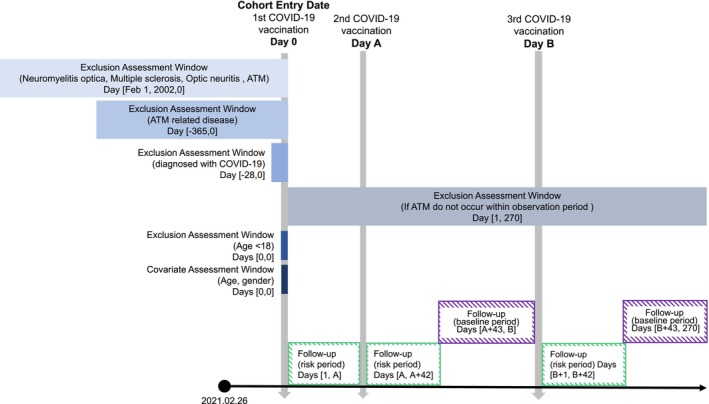
Study diagram for self‐controlled case series analysis. The cohort entry date was the day of first COVID‐19 vaccination. We excluded people who were clinical trial subjects, vaccinated abroad, had incomplete record, or did not reside in South Korea. Also, we excluded individuals who had a history of acute transverse myelitis (ATM) or ATM‐related disease or had previous COVID‐19 infection. The follow‐up period was 1–270 days after the first vaccination, and patients who were not diagnosed with ATM during this observation period were excluded. The risk period was defined as the 42 days following COVID‐19 vaccination. If a participant was vaccinated during the risk period, we extended the days of risk period.

The observation period was 270 days after the first COVID‐19 vaccine dose (Figure 1). The risk period was defined as 1–42 days following each dose, considering the period during which ATM is likely to occur following exposure [21]. The baseline period was defined as the duration within the observation period during which the risk from vaccination is not expected to influence outcomes, excluding the day of subsequent dose administration and the risk period. If an individual received a subsequent dose of the vaccine within the risk period, the initial risk period ended on the day before the subsequent vaccination, and a new risk period started from the day of the subsequent vaccination, extending for 42 days. The end of the follow‐up date was defined as the earlier date of death or 270 days following the first vaccination.

### Statistical analysis

We described the characteristics of the study participants, who were diagnosed with ATM after receiving the COVID‐19 vaccination. We examined age, sex, months of the first vaccination, health insurance type, type of COVID‐19 vaccine for each dose, vaccine product immediately preceding to initial ATM diagnosis, vaccination status, and the Charlson comorbidity index (CCI) score. We estimated the incidence rate ratios (IRRs) and 95% confidence interval (CI) of ATM following COVID‐19 vaccination by comparing the risk period to the baseline period with a conditional Poisson regression model. The baseline period represents the non‐risk period within the observation period and is defined with a conditional Poisson regression model. Subgroup analyses were conducted by gender, age group (18–29, 30–49, 50–64, 65–74, and over 75), vaccine product immediately preceding to initial ATM diagnosis, and CCI score (0–5 and over 5).

We performed several sensitivity analyses by changing the risk period and the observation period. The risk period was modified to 1–21 days or 1–28 days. In addition, the observation period was altered to 90 days (to include people who received the first COVID‐19 vaccination from February 26, 2021, to June 2, 2022), and again to 180 days (to include people who received the first COVID‐19 vaccination from February 26, 2021, to March 4, 2022) It is to cover a larger number of study subjects, considering that ATM is a rare disease. All statistical analyses were conducted using SAS Enterprise Guide 8.2 (SAS Institute, Cary, NC) and a two‐sided p‐value of less than 0.05 was considered statistically significant.

### Literature review

A literature review was performed for all English‐language journals including OVID‐MEDLINE, EMBASE, Cochrane CENTRAL and KoreaMed published from January 1, 2021, to September 30, 2023, utilizing keywords, “COVID‐19” OR “novel coronavirus 2019” OR “2019 coronavirus” OR “2019‐nCoV” OR “corona‐19” AND “ATM” OR “transverse myelitis” OR “myelitis” OR “transverse myelopathy” OR “myelopathy.” ATM articles related to multiple sclerosis (MS), neuromyelitis optica spectrum disorder (NMOSD), optic neuritis, myelin oligodendrocyte glycoprotein antibody disease (MOGAD), ADEM, positive anti‐aquaporin‐4 antibody, and positive myelin oligodendrocyte glycoprotein antibodies were excluded after searching. As a result, we were able to find several studies which indicate the causality between the COVID‐19 vaccination and ATM.

## RESULTS

**FIGURE 2:**
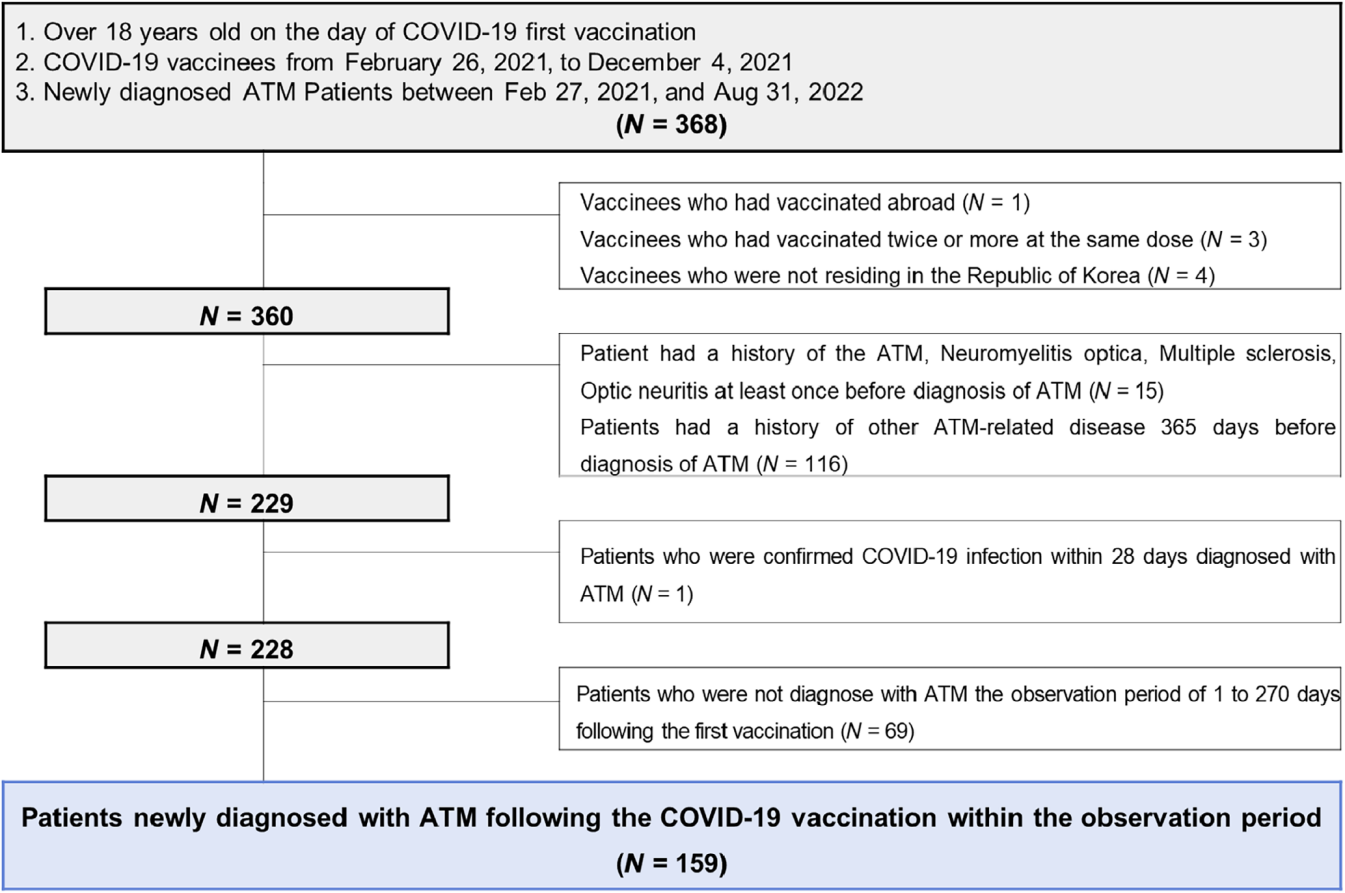
Study flow chart for patients newly diagnosed with acute transverse myelitis (ATM) following the COVID‐19 vaccination.

The total number of COVID‐19 vaccination between February 26, 2021, and August 31, 2022 was 128,223,471. We identified a total of 368 individuals aged 18 or older who received their first COVID‐19 vaccination and were diagnosed with ATM during the study period (Figure 2). Among the 159 eligible patients included in the analysis, 82 (51.6%) were male and the mean age was 55.4 years (±17.4 years) (Table 1). During the 1–42 days post‐vaccination risk period for COVID‐19, 85 cases were identified, while 74 cases were found in the corresponding baseline period. Among the ATM patients found in the risk period, 16 patients (18.8%) had their first COVID‐19 vaccination prior to the ATM diagnosis, 28 patients (32.9%) were vaccinated up to two doses prior to the ATM diagnosis, and 41 patients (48.2%) received three doses before the ATM diagnosis. Among the ATM patients found in the baseline period, seven patients (9.5%) completed their first COVID‐19 vaccination prior to the ATM diagnosis, 38 patients (51.4%) received the second dose before the ATM diagnosis, and 29 patients (39.2%) received three doses prior to ATM diagnosis. There was no statistically significant difference in the demographic and vaccination characteristics of the patients in the risk and baseline periods.

**TABLE 1:** Characteristics of patients who received COVID‐19 vaccination and had a diagnosis of acute transverse myelitis identified during the risk or baseline periods through self‐controlled case series analysis.

Characteristics	Total	Cases in risk period	Cases in baseline period	*P*
	*N* (%)	*N* (%)	*N* (%)	
Total	159 (100%)	74 (100.0%)	85 (100.0%)	
Age (years)				0.690
Mean ± SD	55.4 ± 17.4			
18–29	16 (10.1%)	6 (7.1%)	10 (13.5%)	
30–49	42 (26.4%)	23 (27.1%)	19 (25.7%)	
50–64	44 (27.7%)	26 (30.6%)	18 (24.3%)	
65–74	32 (20.1%)	17 (20.0%)	15 (20.3%)	
Over 75	25 (15.7%)	13 (15.3%)	12 (16.2%)	
Gender				0.959
Male	82 (51.6%)	44 (51.8%)	38 (51.4%)	
Female	77 (48.4%)	41 (48.2%)	36 (48.7%)	
Month of first vaccination				0.713
March 2021	4 (2.5%)	1 (1.2%)	3 (4.1%)	
April 2021	29 (18.2%)	16 (18.8%)	13 (17.6%)	
May 2021	18 (11.3%)	8 (9.4%)	10 (13.5%)	
June 2021	40 (25.2%)	22 (25.9%)	18 (24.3%)	
July 2021	18 (11.3%)	9 (10.6%)	9 (12.2%)	
August 2021	26 (16.4%)	17 (20%)	9 (12.2%)	
September 2021	20 (12.6%)	11 (12.9%)	9 (12.2%)	
October 2021	2 (1.3%)	1 (1.2%)	1 (1.4%)	
November 2021	2 (1.3%)	0 (0.0%)	2 (2.7%)	
Insurance type				1.000
Health insurance	154 (96.9%)	82 (96.5%)	72 (97.3%)	
Medical aid	5 (3.1%)	3 (3.5%)	2 (2.7%)	
Vaccine type (first dose)^a^				0.743
BNT162b2	72 (45.3%)	37 (43.5%)	35 (47.3%)	
ChAdOx1	65 (40.9%)	35 (41.2%)	30 (40.5%)	
mRNA‐1273	17 (10.7%)	11 (12.9%)	6 (8.1%)	
Ad26.COV2.S	5 (3.1%)	2 (2.4%)	3 (4.1%)	
Vaccine type (second dose)				0.536
BNT162b2	83 (52.2%)	41 (48.2%)	42 (56.8%)	
ChAdOx1	33 (20.8%)	17 (20.0%)	16 (21.6%)	
mRNA‐1273	15 (9.4%)	9 (10.6%)	6 (8.1%)	
NVX‐CoV2373	0 (0.0%)	0 (0.0%)	0 (0.0%)	
Vaccine type (third dose)				0.214
BNT162b2	50 (31.4%)	29 (34.1%)	21 (28.4%)	
ChAdOx1	0 (0.0%)	0 (0.0%)	0 (0%)	
mRNA‐1273	18 (11.3%)	12 (14.1%)	6 (8.1%)	
Ad26.COV2.S	0 (0.0%)	0 (0.0%)	0 (0.0%)	
NVX‐CoV2373	2 (1.3%)	0 (0.0%)	2 (2.7%)	
Vaccine product immediately preceding to initial acute transverse myelitis				0.536
BNT162b2	85 (53.5%)	41 (48.2%)	44 (59.5%)	
ChAdOx1	45 (28.3%)	27 (31.8%)	18 (24.3%)	
mRNA‐1273	25 (15.7%)	15 (17.7%)	10 (13.5%)	
Ad26.COV2.S	4 (2.5%)	2 (2.4%)	2 (2.7%)	
NVX‐CoV2373	0 (0.0%)	0 (0.0%)	0 (0.0%)	
Number of doses received prior to initial acute transverse myelitis				0.042
1	23 (14.5%)	16 (18.8%)	7 (9.5%)	
2	66 (41.5%)	28 (32.9%)	38 (51.4%)	
3	70 (44.0%)	41 (48.2%)	29 (39.2%)	
Charlson comorbidity index score				0.854
0–5	27 (17.0%)	14 (16.5%)	13 (17.6%)	
+ 5	132 (83.0%)	71 (83.5%)	61 (82.4%)	

^a^ Individuals who had received at least the first dose of the COVID‐19 vaccine were included, which means individuals without a second or third vaccination were also part of the analysis. The percentage of vaccine types was calculated based on the total number of patients included in the study.

The IRR of ATM in 1–42 days was significantly higher compared with the baseline period (IRR: 2.44, 95% CI: 1.79 to 3.34) (Table 2). Subgroup analyses showed a significantly increased risk across various gender, age, vaccination brand categories, and CCI score (Table 3). The IRR of the subgroup analysis for the type of vaccine administered before the ATM diagnosis was as follows: 3.33 (95% CI: 1.82–6.08) for ChAdOx1; 2.03 (95% CI: 1.34–3.09) for BNT162b2; 2.57 (95% CI: 1.14–5.97) for mRNA‐1273; and 3.33 (95% CI: 0.30–36.44) for Ad26.COV2.S. This trend was consistent in sensitivity analyses (risk window: 1–21 days IRR: 1.96, 95% CI: 1.39–2.78; 1–28 days IRR: 2.15, 95% CI: 1.56–2.97), suggesting an elevated risk of ATM following vaccination. The results of the sensitivity analysis, in which the observation period was altered, indicated an elevated risk (Tables S2–S10).

**TABLE 2:** Risk of acute transverse myelitis following COVID‐19 vaccination in self‐controlled case series analysis (observation period: 270 days following the first dose).

Length of risk period after each dose	Number of cases		IR per person‐year		IRR (95% CI)
	Risk period	Baseline period	Risk period	Baseline period	
1–42 days	85	74	2.31	0.96	2.41 (1.76–3.30)
1–28 days	60	98	2.36	1.11	2.13 (1.54–2.94)
1–21 days	45	113	2.32	1.19	1.94 (1.37–2.76)

Abbreviations: CI, confidence interval; IR, incidence rate; IRR, incidence rate ratio.

**TABLE 3:** Subgroup analysis for the risk of acute transvers myelitis patients following COVID‐19 vaccination in self‐controlled case series analysis (observation period: 270 days following the first dose).

Subgroup	No. of cases		IR per person‐year		IRR (95% CI)
	Risk period	Baseline period	Risk period	Baseline period	
Age (years)					
18–29	6	10	1.88	1.18	1.59 (0.60–4.19)
30–49	23	19	2.48	0.89	2.77 (1.47–5.22)
50–64	26	18	2.37	0.87	2.74 (1.49–5.03)
65–74	17	15	2.15	0.98	2.18 (1.08–4.42)
≥75	13	12	2.36	1.03	2.29 (1.07–4.89)
Gender					
Male	44	38	2.33	0.97	2.40 (1.53–3.76)
Female	41	36	2.28	0.94	2.43 (1.57–3.75)
Vaccine product immediately preceding to initial acute transverse myelitis					
BNT162b2	41	44	2.06	1.23	1.99 (1.30–3.03)
ChAdOx1	27	18	2.70	0.82	3.31 (1.81–6.05)
mRNA‐1273	15	10	2.25	0.87	2.57 (1.14–5.79)
Ad26.COV2.S	2	2	2.97	0.89	3.33 (0.30–36.44)
NVX‐CoV2373	0	0	N/A	N/A	N/A
Charlson comorbidity index score					
0–5	14	13	2.11	1.01	2.10 (0.99–4.42)
+ 5	71	61	2.35	0.95	2.48 (1.76–3.51)

Abbreviations: CI, confidence interval; IR, incidence rate; IRR, incidence rate ratio.

## DISCUSSION

This nationwide population‐based SCCS study identified a significant increase in the incidence of ATM within 1–42 days following COVID‐19 vaccination. This elevated risk was consistent across various subgroups, including different types of vaccine products and patients with CCI scores >5. Our findings contribute to the ongoing debate on the safety profiles of COVID‐19 vaccines by providing evidence of potential neurological complications.

We aimed to identify the demographic and clinical characteristics of ATM that developed after COVID‐19 vaccination. For a comprehensive understanding, we undertook a literature review focused on existing literature regarding the occurrence of ATM following COVID‐19 vaccination.

Walker et al. conducted a study with SCCS, the same design employed in our study and used UK's records for primary care. There was no clear evidence of an association between the respective ChAdOx1 and BNT162b2 vaccinations and TM [22]. The incidence and relative risk of TM are too low to detect an association in the general adult population. In a population‐based study that compared historical rates with the SCCS analysis based on the data from primary care records in both the UK and Spain, the incidence of TM, defined as occurring within 21 days after the first vaccination, was less than five events [23]. These discrepancies may arise from different study populations, vaccine types, and follow‐up durations.

In a retrospective cohort study using VigiBase, the WHO's pharmacovigilance database, a disproportionality analysis was conducted as an information component. The analysis revealed that TM was significantly associated with mRNA‐based and vector‐based COVID‐19 vaccines. However, it is important to note that evidence for a diagnosis of TM is still lacking and some TM cases may include other neurological syndromes. Additionally, the possibility that TM cases are related to SARS‐CoV‐2 infection itself cannot be ruled out [13]. In an open‐label, phase 3b implementation study for South Africa registered in the National Registry, known as the Sisonke study, adverse events (AEs) were monitored at the vaccination site through self‐reporting via text message after vaccination. TM incidence per 100,000 person‐years was 2.11 (95% CI: 0.53–8.44) and O/E ratio was only 0.08 (95% CI: 0.01–0.27) [24]. Kim et al. used disproportionate analysis to rapidly detect relevant safety signals and compared the relative risks associated with diverse ranges of vaccines. Compared with the influenza vaccine, the mRNA COVID‐19 vaccine did not show a significant difference in the incidence of myelitis [25]. Our literature review found limited conclusive evidence directly linking vaccines to ATM, suggesting that reported cases could include misdiagnoses or unrelated neurological symptoms. The variability in the diagnostic criteria for ATM and the challenges in distinguishing it from other neurological conditions without extensive investigations could also contribute to inconsistencies across studies.

ATM refers to a heterogeneous group of inflammatory spinal cord disorders that may occur independently or may be part of either MS, NMO, systemic connective tissue disease, infectious disease, radiation, or malignancy. The pathogenesis of TM appears to be immune‐mediated from infection, para‐infection, and autoimmune disease [8]. Post‐vaccination TM may be associated with various types of immune pathways [6]. Infectious agents and vaccine adjuvants are thought to induce autoimmunity in several ways, and there are several proposed mechanisms [21]. First, molecular mimicry is a concept associated with microbial epitopes that share striking similarities with the host's antigens [13, 26], and allows lymphocytes activated by infection to cross‐react with self‐antigens [27]. Second, the initial immune response to an acute infection is highly specific, but can be extended to other epitopes of the new pathogen by epitope spreading [28]. Third, the bystander activation theory holds that the inflammatory cascade induces autoimmunity by stimulating autoreactive immune cells, and that systemic inflammation also causes dysfunction of the blood–brain barrier, allowing autoreactive cells access to the central nervous system [29]. Finally, chronic infection causes polyclonal B‐ and T‐cell expansion, a process that can result in the production of autoantibodies that contribute to the development of autoimmunity [30]. Various immunopathological mechanisms have been proposed to explain the association between vaccines and ATM. These include molecular mimicry, where vaccine components mimic body tissues leading to an autoimmune response, and bystander activation, where the vaccine‐induced immune response inadvertently activates self‐reactive immune cells. Such mechanisms could vary significantly between individuals due to genetic differences or pre‐existing health conditions, potentially explaining the differences in vaccine reactions among populations. We considered the timing of TM events in relation to the vaccination. It is generally accepted that post‐vaccinal CNS demyelination occurs after 7 days post‐vaccination [31]. We analyzed the number of TM cases that occurred within the first 1–6 days post‐vaccination. Our findings showed that 5 TM cases were diagnosed within this period. This suggests that while some instances of TM do occur soon after vaccination, the majority of TM cases occur within the expected timeframe of post‐vaccinal CNS demyelination, supporting the validity of our defined risk period.

However, these mechanisms may provide only a partial explanation for the pathophysiology of ATM caused by vaccines containing viral adjuvants capable of mediating immune responses that target spinal cord. For vector‐based COVID‐19 vaccines, the use of chimpanzee adenovirus vectors has been proposed to play a role in inducing autoimmunity [21]. On the contrary, in the case of mRNA‐based COVID‐19 vaccines, an immunological reaction between SARS‐CoV‐2 spike protein antibodies and tissue proteins may contribute to the development of demyelinating autoimmune disease [32]. Studies on the immunopathogenesis of ATM have also highlighted the role of interleukins IL‐6 and IL‐17 [33]. However, a clear causal relationship between SARS‐CoV‐2 vaccine and ATM still needs further research.

Although the overall incidence of ATM is still rare, our study showed an increased risk of ATM following COVID‐19 vaccination. Previous literature has not drawn conclusions as clear as our findings because most earlier studies relied on voluntary AE reporting data from countries such as the United States and the UK. However, these reports often have problems of underreporting, and inconsistencies in data quality, which can lead to bias. Additionally, it can cause disparities when compared to studies using insurance claims data from larger populations.

Nonetheless, our findings are not entirely free from the two sources of bias: underestimation and overestimation of the results. Given that the patient groups were defined based on the diagnosis code claimed to the NHIS, it is possible that patients could have been assigned a specific diagnosis even when they simply access medical service only to see if they have disease. In addition, the growing awareness among experts and the public, driven by broadcasting and media, regarding a potential connection between COVID‐19 vaccination and ATM may introduce a potential detection bias, as more people may have sought medical help and received treatment for similar symptoms. This increased awareness might also lead to misdiagnoses of similar conditions, such as neuromyelitis optical and multiple sclerosis, due to heightened vigilance.

A SCCS design is its lack of an external control group. This methodology capitalizes on each participant serving as their own control, effectively managing within‐person variability. However, it does not facilitate comparisons with an unexposed population. This absence can complicate the ability to fully isolate the impact of external factors or confounding variables on study outcomes. Therefore, it is crucial to interpret findings with caution, and such limitations must be explicitly acknowledged during the analysis phase to ensure a balanced understanding of the results.

Among patients with confirmed ATM, we attempted to exclude similar central nervous system inflammatory demyelinating diseases such as NMOSD, MS, or MOGAD. However, the initial identification of ATM cases was based on the health insurance claims database, utilizing ICD‐10 and procedure codes. This approach may have introduced potential biases due to misclassification, as the database lacks detailed procedure and treatment information. Given that ATM can be an initial symptom of NMOSD, MS, or MOGAD, some cases require continuous follow‐up to determine their progression. We cannot rule out the possibility that some misclassified patients were included in this study. Furthermore, our reliance on ICD‐10 codes for diagnosing ATM introduces a risk of misclassification, a common issue in large database studies. This limitation is particularly significant when comparing our findings to studies that utilize primary clinical assessments for diagnosis. Standardizing diagnostic criteria across studies could help reduce such discrepancies and provide a more accurate assessment of ATM incidence following vaccination.

Furthermore, our analysis benefits from South Korea's robust healthcare infrastructure, which boasts a high density of MRI machines. According to the Organization for Economic Co‐operation and Development (OECD) health statistics in 2023, Korea ranked first among OECD countries in terms of the number of outpatient treatments per capita, indicating high accessibility of medical care for domestic patients. Specifically, South Korea has a high number of MRI machines, with 30.1 units per million people, 1.8 times more than the OECD average of 17.0 [34]. This number increased from 24.4 units in 2013 to 30.1 units in 2018, surpassing the OECD's average growth rate. The widespread availability of MRI equipment, which is rising across all types of medical institutions except for general hospitals, likely contributes to a higher detection rate of ATM compared with regions with less diagnostic capability. This could potentially explain some of the discrepancies observed between our results and those from studies conducted in other settings. The expanded availability of MRI and frequent MRI tests have significantly improved diagnostic sensitivity, enabling more reliable identification of ATM patients.

The consistent results of sensitivity analyses increased the robustness of our findings. The utilization of a nationwide database in our study allowed us to comprehensively investigate this rare AE, thereby increasing the generalizability of our findings. The SCCS design was particularly advantageous in this study where non‐exposure controls were insufficient because of the mass vaccination situation. Since the study covers a period of approximately 18 months following the vaccine introduction, it incorporates both initial post‐vaccination data and subsequent information. This comprehensive timeframe lends confidence to the stability of the observed results.

## CONCLUSION

Our findings indicate an increased risk of ATM following COVID‐19 vaccination regardless of whether it is a viral vector and mRNA vaccine. Physicians should exercise caution regarding the possibility of ATM following vaccination, enabling early diagnosis and appropriate management. Further research is needed to understand the mechanisms behind this association and to guide post‐vaccination monitoring and treatment strategies.

## AUTHOR CONTRIBUTIONS

**Eunsun Lim:** Writing – original draft; conceptualization; methodology; formal analysis; visualization; investigation; data curation; software. **Yoo Hwan Kim:** Writing – original draft; conceptualization; investigation; validation; methodology. **Na‐Young Jeong:** Conceptualization; methodology; formal analysis; investigation; data curation; software. **Su‐Hyun Kim:** Writing – review and editing; visualization; methodology; validation; investigation. **Heehyun Won:** Writing – review and editing; visualization; investigation. **Jong‐Seok Bae:** Project administration; writing – review and editing; conceptualization; supervision; investigation. **Nam‐Kyong Choi:** Project administration; writing – review and editing; conceptualization; supervision; investigation.

## CONFLICT OF INTEREST STATEMENT

NK having contract with Moderna for ‘Post–marketing safety of Spikevax vaccine in South Korea’, but analyses were finished before the contract and Moderna had no role in the design and conduct of the study.

## Supporting information


**Appendix S1.** Supporting Information.

**Appendix S2.** Supporting Information.

## Data Availability

The data that support the findings of this study are available from NHIS. Restrictions apply to the availability of these data, which were used under license for this study. Data are available from the author(s) with the permission of NHIS.
