# Supplementary material for: The association between acute transverse myelitis and COVID‐19 vaccination in Korea: Self‐controlled case series study
Source: Eur J Neurol. 2024 Dec 30;32(1):e70020. doi: 10.1111/ene.70020 (PMC11683473; doi:10.1111/ene.70020)
Supplement: Supplementary file 2 — Appendix S2. Supporting Information. [file ENE-32-e70020-s001.docx]

Supplementary table 1. Diagnosis or procedure code used in the study

| **Category** | **ICD-10**  **or procedure code** | **Description** |
| --- | --- | --- |
| Diagnosis code related to acute transvers myelitis | G37.3 | Acute transverse myelitis in demyelinating disease of central nervous system |
| Screening tests or procedure code related to acute transverse myelitis | D0327 | Body Fluid-Examination(CSF, Ascites, Pleural Fluid, Joint Fluid) (Color, Gravity, Cell Count, Differential Count, pH) |
|  | HE109 | Cervical Spine MRI |
|  | HE110 | Thoracic Spine MRI |
|  | HE111 | Lumbosacral Spine MRI |
|  | HE113 | MRI (Spine, Lumbosacral, Thoracic) |
|  | HE114 | MRI (Spine, Myelogram, Cervical, Lumbosacral) |
|  | HE209 | Cervical Spine MRI |
|  | HE210 | Thoracic Spine MRI |
|  | HE211 | Lumbosacral Spine MRI |
| History of disease prior to 365 days of onset of acute transverse myelitis | G37.3 | Acute transverse myelitis in demyelinating disease of central nervous system |
|  | G04.0 | Acute disseminated encephalitis |
|  | G04.8 | Other encephalitis, myelitis and encephalomyelitis |
|  | G04.9 | Encephalitis, myelitis and encephalomyelitis, unspecified |
|  | G05.* | Encephalitis, myelitis and encephalomyelitis in diseases classified elsewhere |
| History of disease prior to onset of acute transverse myelitis | G36.0 | Neuromyelitis optica |
|  | G35 | Multiple sclerosis |
|  | H46 | Optic neuritis |
|  | H48.1 | Retrobulbar neuritis in diseases classified elsewhere |

Abbreviation: ICD-10 code, International Classification of Diseases, Tenth Revision; CSF, Cerebrospinal fluid; MRI, Magnetic resonance imaging.

Supplementary table 2. Characteristics of patients who received COVID-19 vaccination within 90 days and had a diagnosis of acute transverse myelitis during the risk or baseline periods for self-controlled case series analysis (Risk period: 1-21)

| **Characteristics** | **Total** | | **Cases in risk period** | | **Cases in baseline period** | | *P* |
| --- | --- | --- | --- | --- | --- | --- | --- |
|  | **N (%)** | | **N (%)** | | **N (%)** | |  |
| **Total** | 80 (100%) | | 35 (100.0%) | | 45 (100.0%) | |  |
| **Age (years)** | | | | | | | 0.853 |
| 18–29 | 10 (12.5%) | | 6 (17.1%) | | 4 (8.9%) | |  |
| 30–49 | 24 (30.0%) | | 10 (28.6%) | | 14 (31.1%) | |  |
| 50–64 | 17 (21.3%) | | 7 (20%) | | 10 (22.2%) | |  |
| 65–74 | 18 (22.5%) | | 7 (20%) | | 11 (24.4%) | |  |
| Over 75 | 11 (13.7%) | | 5 (14.3%) | | 6 (13.3%) | |  |
| **Gender** |  |  | |  | |  | 0.535 |
| Male | 38 (47.5%) | | 18 (51.4%) | | 20 (44.4%) | |  |
| Female | 42 (52.5%) | | 17 (48.6%) | | 25 (55.6%) | |  |
| **Month of first vaccination** |  |  | |  | |  | 0.723 |
| March, 2021 | 1 (1.3%) | | 0 (0%) | | 1 (2.2%) | |  |
| April, 2021 | 14 (17.5%) | | 5 (14.3%) | | 9 (20%) | |  |
| May, 2021 | 7 (8.7%) | | 4 (11.4%) | | 3 (6.7%) | |  |
| June, 2021 | 22 (27.5%) | | 9 (25.7%) | | 13 (28.9%) | |  |
| July, 2021 | 8 (10.0%) | | 3 (8.6%) | | 5 (11.1%) | |  |
| August, 2021 | 11 (13.7%) | | 5 (14.3%) | | 6 (13.3%) | |  |
| September, 2021 | 12 (15.0%) | | 6 (17.1%) | | 6 (13.3%) | |  |
| October, 2021 | 1 (1.3%) | | 1 (2.9%) | | 0 (0%) | |  |
| November, 2021 | 2 (2.5%) | | 0 (0%) | | 2 (4.4%) | |  |
| **Insurance type** |  |  | |  | |  | 0.438 |
| Health insurance | 79 (98.7%) | | 34 (97.1%) | | 45 (100%) | |  |
| Medical aid | 1 (1.3%) | | 1 (2.9%) | | 0 (0%) | |  |
| **Vaccine type (first dose)** |  |  | |  | |  | 0.526 |
| BNT162b2 | 33 (41.3%) | | 14 (39.9%) | | 22 (48.9%) | |  |
| ChAdOx1 | 34 (42.5%) | | 15 (42.9%) | | 18 (40%) | |  |
| mRNA-1273 | 8 (10.0%) | | 5 (14.3%) | | 3 (6.7%) | |  |
| Ad26.COV2.S | 3 (3.7%) | | 1 (2.9%) | | 2 (4.4%) | |  |
| **Vaccine type (second dose)** |  |  | |  | |  | 0.235 |
| BNT162b2 | 35 (43.8%) | | 17 (48.6%) | | 18 (40%) | |  |
| ChAdOx1 | 12 (15.0%) | | 5 (14.3%) | | 7 (15.6%) | |  |
| mRNA-1273 | 7 (8.7%) | | 5 (14.3%) | | 2 (4.4%) | |  |
| NVX-CoV2373 | 0 (0.0%) | | 0 (0%) | | 0 (0%) | |  |
| **Vaccine type (third dose)** |  |  | |  | |  | 0.613 |
| BNT162b2 | 20 (25.0%) | | 7 (20%) | | 13 (28.9%) | |  |
| ChAdOx1 | 0 (0.0%) | | 0 (0.0%) | | 0 (0.0%) | |  |
| mRNA-1273 | 4 (5.0%) | | 2 (5.7%) | | 2 (4.4%) | |  |
| Ad26.COV2.S | 0 (0.0%) | | 0 (0.0%) | | 0 (0.0%) | |  |
| NVX-CoV2373 | 0 (0.0%) | | 0 (0.0%) | | 0 (0.0%) | |  |
| **Vaccine product immediately preceding to initial acute transverse myelitis** | | | | | | | 0.213 |
| BNT162b2 | 35 (43.7%) | | 17 (48.6%) | | 18 (40%) | |  |
| ChAdOx1 | 32 (40.0%) | | 10 (28.6%) | | 22 (48.9%) | |  |
| mRNA-1273 | 9 (11.3%) | | 6 (17.1%) | | 3 (6.7%) | |  |
| Ad26.COV2.S | 3 (3.8%) | | 1 (2.9%) | | 2 (4.4%) | |  |
| NVX-CoV2373 | 0 (0.0%) | | 0 (0.0%) | | 0 (0.0%) | |  |
| **Number of doses received prior to initial acute transverse myelitis** | | | | | | | 0.100 |
| 1 | 23 (28.7%) | | 7 (20%) | | 16 (35.6%) | |  |
| 2 | 33 (41.3%) | | 19 (54.3%) | | 14 (31.1%) | |  |
| 3 | 24 (30.0%) | | 9 (25.7%) | | 15 (33.3%) | |  |
| **Charlson comorbidity index score** | | | | | | | 0.088 |
| 0–5 | 14 (17.5%) | | 9 (25.7%) | | 5 (11.1%) | |  |
| + 5 | 66 (82.5%) | | 26 (74.3%) | | 40 (88.9%) | |  |

* Individuals who had received at least the first dose of the COVID-19 vaccine were included, which means individuals without a second or third vaccination were also part of the analysis. The percentage of vaccine types was calculated based on the total number of patients included in the study.

Supplementary table 3. Characteristics of patients who received COVID-19 vaccination within 90 days and had a diagnosis of acute transverse myelitis during the risk or the baseline periods for self-controlled case series analysis (Risk period: 1-28)

| **Characteristics** | **Total** | | **Cases in risk period** | | **Cases in baseline period** | | *P* |
| --- | --- | --- | --- | --- | --- | --- | --- |
|  | **N (%)** | | **N (%)** | | **N (%)** | |  |
| **Total** | 80 (100.0%) | | 48 (100.0%) | | 32 (100.0%) | |  |
| **Age (years)** | | | | | | | 0.801 |
| 18–29 | 10 (12.5%) | | 7 (14.6%) | | 3 (9.4%) | |  |
| 30–49 | 24 (30.0%) | | 12 (25%) | | 12 (37.5%) | |  |
| 50–64 | 17 (21.3%) | | 11 (22.9%) | | 6 (18.7%) | |  |
| 65–74 | 18 (22.5%) | | 11 (22.9%) | | 7 (21.9%) | |  |
| Over 75 | 11 (13.8%) | | 7 (14.6%) | | 4 (12.5%) | |  |
| **Gender** |  |  | |  | |  | 0.583 |
| Male | 38 (47.5%) | | 24 (50%) | | 14 (43.8%) | |  |
| Female | 42 (52.5%) | | 24 (50%) | | 18 (56.2%) | |  |
| **Month of first vaccination** |  |  | |  | |  | 0.816 |
| March, 2021 | 1 (1.3%) | | 0 (0%) | | 1 (3.1%) | |  |
| April, 2021 | 14 (17.5%) | | 9 (18.7%) | | 5 (15.6%) | |  |
| May, 2021 | 7 (8.7%) | | 4 (8.3%) | | 3 (9.4%) | |  |
| June, 2021 | 22 (27.5%) | | 13 (27.1%) | | 9 (28.1%) | |  |
| July, 2021 | 8 (10.0%) | | 5 (10.4%) | | 3 (9.4%) | |  |
| August, 2021 | 11 (13.8%) | | 7 (14.6%) | | 4 (12.5%) | |  |
| September, 2021 | 12 (15.0%) | | 7 (14.6%) | | 5 (15.6%) | |  |
| October, 2021 | 1 (1.2%) | | 1 (2.1%) | | 0 (0.0%) | |  |
| November, 2021 | 2 (2.5%) | | 0 (0%) | | 2 (6.3%) | |  |
| **Insurance type** |  |  | |  | |  | 1.000 |
| Health insurance | 79 (98.8%) | | 47 (97.9%) | | 32 (100.0%) | |  |
| Medical aid | 1 (1.2%) | | 1 (2.1%) | | 0 (0.0%) | |  |
| **Vaccine type (first dose)** |  |  | |  | |  | 0.602 |
| BNT162b2 | 35 (43.8%) | | 20 (37.5%) | | 15 (46.9%) | |  |
| ChAdOx1 | 34 (42.5%) | | 21 (43.8%) | | 13 (40.7%) | |  |
| mRNA-1273 | 8 (10.0%) | | 6 (12.5%) | | 2 (6.2%) | |  |
| Ad26.COV2.S | 3 (3.7%) | | 1 (2.2%) | | 2 (6.2%) | |  |
| **Vaccine type (second dose)** |  |  | |  | |  | 0.325 |
| BNT162b2 | 35 (43.8%) | | 18 (37.5%) | | 13 (40.6%) | |  |
| ChAdOx1 | 12 (15.0%) | | 21 (43.8%) | | 3 (9.4%) | |  |
| mRNA-1273 | 7 (8.7%) | | 6 (12.5%) | | 2 (6.3%) | |  |
| NVX-CoV2373 | 0 (0.0%) | | 1 (2.2%) | | 0 (0.0%) | |  |
| **Vaccine type (third dose)** |  |  | |  | |  | 0.465 |
| BNT162b2 | 20 (25.0%) | | 14 (29.2%) | | 6 (18.8%) | |  |
| ChAdOx1 | 0 (0.0%) | | 0 (0.0%) | | 0 (0.0%) | |  |
| mRNA-1273 | 4 (5.0%) | | 3 (6.3%) | | 1 (3.1%) | |  |
| Ad26.COV2.S | 0 (0.0%) | | 0 (0.0%) | | 0 (0.0%) | |  |
| NVX-CoV2373 | 20 (25.0%) | | 14 (29.2%) | | 6 (18.8%) | |  |
| **Vaccine product immediately preceding to initial acute transverse myelitis** | | | | | | | 0.575 |
| BNT162b2 | 35 (43.8%) | | 20 (41.7%) | | 15 (46.9%) | |  |
| ChAdOx1 | 32 (40.0%) | | 19 (39.6%) | | 13 (40.7%) | |  |
| mRNA-1273 | 9 (11.4%) | | 7 (14.6%) | | 2 (6.2%) | |  |
| Ad26.COV2.S | 3 (3.8%) | | 1 (2.1%) | | 2 (6.2%) | |  |
| NVX-CoV2373 | 0 (0%) | | 0 (0.0%) | | 0 (0.0%) | |  |
| **Number of doses received prior to initial acute transverse myelitis** | | | | | | | 0.272 |
| 1 | 23 (28.7%) | | 11 (22.9%) | | 12 (37.5%) | |  |
| 2 | 33 (41.3%) | | 20 (41.7%) | | 13 (40.6%) | |  |
| 3 | 24 (30.0%) | | 17 (35.4%) | | 7 (21.9%) | |  |
| **Charlson comorbidity index score** | | | | | | | 0.718 |
| 0–5 | 14 (17.5%) | | 9 (18.8%) | | 5 (15.6%) | |  |
| + 5 | 66 (82.5%) | | 39 (81.2%) | | 27 (84.4%) | |  |

* Individuals who had received at least the first dose of the COVID-19 vaccine were included, which means individuals without a second or third vaccination were also part of the analysis. The percentage of vaccine types was calculated based on the total number of patients included in the study.

Supplementary table 4. Characteristics of patients who received COVID-19 vaccination within 180 days and had a diagnosis of acute transverse myelitis during the risk or the baseline periods for self-controlled case series analysis (Risk period: 1-21)

| **Characteristics** | **Total** | | **Cases in risk period** | | **Cases in baseline period** | | *P* |
| --- | --- | --- | --- | --- | --- | --- | --- |
|  | **N (%)** | | **N (%)** | | **N (%)** | |  |
| **Total** | 137 (100.0%) | | 43 (100.0%) | | 94 (100.0%) | |  |
| **Age (years)** | | | | | | | 0.962 |
| 18–29 | 16 (11.7%) | | 6 (14.0%) | | 10 (10.6%) | |  |
| 30–49 | 40 (29.2%) | | 12 (27.9%) | | 28 (29.8%) | |  |
| 50–64 | 36 (26.3%) | | 12 (27.9%) | | 24 (25.5%) | |  |
| 65–74 | 26 (19.0%) | | 8 (18.6%) | | 18 (19.1%) | |  |
| Over 75 | 19 (13.9%) | | 5 (11.6%) | | 14 (14.9%) | |  |
| **Gender** |  |  | |  | |  | 0.621 |
| Male | 69 (50.4%) | | 23 (53.5%) | | 46 (48.9%) | |  |
| Female | 68 (49.6%) | | 20 (46.5%) | | 48 (51.1%) | |  |
| **Month of first vaccination** |  |  | |  | |  | 0.735 |
| March, 2021 | 2 (1.5%) | | 0 (0.0%) | | 2 (2.1%) | |  |
| April, 2021 | 22 (16.0%) | | 6 (14.0%) | | 16 (17.0%) | |  |
| May, 2021 | 15 (10.9%) | | 4 (9.3%) | | 11 (11.7%) | |  |
| June, 2021 | 31 (22.6%) | | 11 (25.6%) | | 20 (21.3%) | |  |
| July, 2021 | 17 (12.4%) | | 5 (11.6%) | | 12 (12.8%) | |  |
| August, 2021 | 24 (17.5%) | | 7 (16.4%) | | 17 (18.1%) | |  |
| September, 2021 | 20 (14.6%) | | 7 (16.4%) | | 13 (13.8%) | |  |
| October, 2021 | 2 (1.5%) | | 1 (2.3%) | | 1 (1.1%) | |  |
| November, 2021 | 2 (1.5%) | | 0 (0.0%) | | 2 (2.1%) | |  |
| **Insurance type** |  |  | |  | |  | 1.000 |
| Health insurance | 133 (97.1%) | | 42 (97.7%) | | 91 (96.8%) | |  |
| Medical aid | 4 (2.9%) | | 1 (2.3%) | | 3 (3.2%) | |  |
| **Vaccine type (first dose)** |  |  | |  | |  | 0.646 |
| BNT162b2 | 63 (46.0%) | | 19 (44.2%) | | 44 (46.8%) | |  |
| ChAdOx1 | 52 (38.0%) | | 15 (34.9%) | | 37 (39.4%) | |  |
| mRNA-1273 | 18 (13.1%) | | 8 (18.6%) | | 10 (10.6%) | |  |
| Ad26.COV2.S | 4 (2.9%) | | 1 (2.3%) | | 3 (3.2%) | |  |
| **Vaccine type (second dose)** |  |  | |  | |  | 0.718 |
| BNT162b2 | 71 (51.8%) | | 21 (48.8%) | | 50 (53.2%) | |  |
| ChAdOx1 | 22 (16.1%) | | 7 (16.3%) | | 15 (15.9%) | |  |
| mRNA-1273 | 16 (11.7%) | | 7 (16.3%) | | 9 (9.6%) | |  |
| NVX-CoV2373 | 0 (0.0%) | | 0 (0.0%) | | 0 (0%) | |  |
| **Vaccine type (third dose)** |  |  | |  | |  | 0.707 |
| BNT162b2 | 40 (29.2%) | | 10 (23.3%) | | 30 (31.9%) | |  |
| ChAdOx1 | 0 (0.0%) | | 0 (0.0%) | | 0 (0.0%) | |  |
| mRNA-1273 | 10 (7.3%) | | 5 (11.6%) | | 5 (5.3%) | |  |
| Ad26.COV2.S | 2 (1.5%) | | 0 (0.0%) | | 2 (2.1%) | |  |
| NVX-CoV2373 | 85 (62.0%) | | 28 (65.1%) | | 57 (60.6%) | |  |
| **Vaccine product immediately preceding to initial acute transverse myelitis** | | | | | | | 0.362 |
| BNT162b2 | 73 (53.3%) | | 22 (51.2%) | | 51 (54.3%) | |  |
| ChAdOx1 | 41 (29.9%) | | 11 (25.6%) | | 30 (31.9%) | |  |
| mRNA-1273 | 19 (13.9%) | | 9 (20.9%) | | 10 (10.6%) | |  |
| Ad26.COV2.S | 4 (2.9%) | | 1 (2.3%) | | 3 (3.2%) | |  |
| NVX-CoV2373 | 0 (0.0%) | | 0 (0.0%) | | 0 (0.0%) | |  |
| **Number of doses received prior to initial acute transverse myelitis** | | | | | | | 0.790 |
| 1 | 24 (17.5%) | | 7 (16.3%) | | 17 (18.1%) | |  |
| 2 | 61 (44.5%) | | 21 (48.8%) | | 40 (42.6%) | |  |
| 3 | 52 (38.0%) | | 15 (34.9%) | | 37 (39.4%) | |  |
| **Charlson comorbidity index score** | | | | | | | 0.232 |
| 0–5 | 24 (17.5%) | | 10 (23.3%) | | 14 (14.9%) | |  |
| + 5 | 113 (82.5%) | | 33 (76.7%) | | 80 (85.1%) | |  |

* * Individuals who had received at least the first dose of the COVID-19 vaccine were included, which means individuals without a second or third vaccination were also part of the analysis. The percentage of vaccine types was calculated based on the total number of patients included in the study.

Supplementary table 5. Characteristics of patients who received COVID-19 vaccination within 180 days and had a diagnosis of acute transverse myelitis during the risk or the baseline periods for self-controlled case series analysis (Risk period: 1-28)

| **Characteristics** | **Total** | | **Cases in risk period** | | **Cases in baseline period** | | *P* |
| --- | --- | --- | --- | --- | --- | --- | --- |
|  | **N (%)** | | **N (%)** | | **N (%)** | |  |
| **Total** | 137 (100.0%) | | 58 (100.0%) | | 79 (100.0%) | |  |
| **Age (years)** | | | | | | | 0.811 |
| 18–29 | 16 (11.7%) | | 7 (12.1%) | | 9 (11.4%) | |  |
| 30–49 | 40 (29.2%) | | 14 (24.1%) | | 26 (32.9%) | |  |
| 50–64 | 36 (26.3%) | | 16 (27.6%) | | 20 (25.3%) | |  |
| 65–74 | 26 (19.0%) | | 13 (22.4%) | | 13 (16.4%) | |  |
| Over 75 | 19 (13.8%) | | 8 (13.8%) | | 11 (13.9%) | |  |
| **Gender** |  |  | |  | |  | 0.785 |
| Male | 69 (50.4%) | | 30 (51.7%) | | 39 (49.4%) | |  |
| Female | 68 (49.6%) | | 28 (48.3%) | | 40 (50.6%) | |  |
| **Month of first vaccination** |  |  | |  | |  | 0.693 |
| March, 2021 | 2 (1.5%) | | 0 (0.0%) | | 2 (2.5%) | |  |
| April, 2021 | 22 (16.0%) | | 10 (17.2%) | | 12 (15.3%) | |  |
| May, 2021 | 15 (10.9%) | | 5 (8.6%) | | 10 (12.6%) | |  |
| June, 2021 | 31 (22.6%) | | 16 (27.6%) | | 15 (19.0%) | |  |
| July, 2021 | 17 (12.4%) | | 7 (12.1%) | | 10 (12.6%) | |  |
| August, 2021 | 24 (17.5%) | | 9 (15.5%) | | 15 (19.0%) | |  |
| September, 2021 | 20 (14.6%) | | 8 (13.8%) | | 12 (15.2%) | |  |
| October, 2021 | 2 (1.5%) | | 1 (1.7%) | | 1 (1.3%) | |  |
| November, 2021 | 2 (1.5%) | | 0 (0.0%) | | 2 (2.5%) | |  |
| **Insurance type** |  |  | |  | |  | 0.637 |
| Health insurance | 133 (97.1%) | | 57 (98.3%) | | 76 (96.2%) | |  |
| Medical aid | 4 (2.9%) | | 1 (1.7%) | | 3 (3.8%) | |  |
| **Vaccine type (first dose)** |  |  | |  | |  | 0.491 |
| BNT162b2 | 63 (46.0%) | | 23 (39.7%) | | 40 (50.6%) | |  |
| ChAdOx1 | 52 (37.9%) | | 25 (43.1%) | | 27 (34.2%) | |  |
| mRNA-1273 | 18 (13.1%) | | 9 (15.5%) | | 9 (11.4%) | |  |
| Ad26.COV2.S | 4 (2.9%) | | 1 (1.7%) | | 3 (3.8%) | |  |
| **Vaccine type (second dose)** |  |  | |  | |  | 0.593 |
| BNT162b2 | 71 (51.8%) | | 27 (46.5%) | | 44 (55.7%) | |  |
| ChAdOx1 | 22 (16.0%) | | 12 (20.7%) | | 10 (12.7%) | |  |
| mRNA-1273 | 16 (11.7%) | | 7 (12.1%) | | 9 (11.4%) | |  |
| NVX-CoV2373 | 0 (0.0%) | | 0 (0.0%) | | 0 (0.0%) | |  |
| **Vaccine type (third dose)** |  |  | |  | |  | 0.163 |
| BNT162b2 | 40 (29.2%) | | 18 (31.0%) | | 22 (27.8%) | |  |
| ChAdOx1 | 0 (0.0%) | | 0 (0.0%) | | 0 (0.0%) | |  |
| mRNA-1273 | 10 (7.3%) | | 7 (12.1%) | | 3 (3.8%) | |  |
| Ad26.COV2.S | 2 (1.5%) | | 0 (0.0%) | | 2 (2.5%) | |  |
| NVX-CoV2373 | 85 (62.0%) | | 33 (56.9%) | | 52 (65.8%) | |  |
| **Vaccine product immediately preceding to initial acute transverse myelitis** | | | | | | | 0.265 |
| BNT162b2 | 73 (53.3%) | | 26 (44.8%) | | 47 (59.5%) | |  |
| ChAdOx1 | 41 (29.9%) | | 21 (36.2%) | | 20 (25.3%) | |  |
| mRNA-1273 | 19 (13.9%) | | 10 (17.2%) | | 9 (11.4%) | |  |
| Ad26.COV2.S | 4 (2.9%) | | 1 (1.7%) | | 3 (3.8%) | |  |
| NVX-CoV2373 | 0 (0.0%) | | 0 (0.0%) | | 0 (0.0%) | |  |
| **Number of doses received prior to initial acute transverse myelitis** | | | | | | | 0.406 |
| 1 | 24 (17.5%) | | 11 (19.0%) | | 13 (16.5%) | |  |
| 2 | 61 (44.5%) | | 22 (37.9%) | | 39 (49.4%) | |  |
| 3 | 52 (38.0%) | | 25 (43.1%) | | 27 (34.2%) | |  |
| **Charlson comorbidity index score** | | | | | | | 0.942 |
| 0–5 | 24 (17.5%) | | 10 (17.2%) | | 14 (17.7%) | |  |
| + 5 | 113 (82.5%) | | 48 (82.8%) | | 65 (82.3%) | |  |

* Individuals who had received at least the first dose of the COVID-19 vaccine were included, which means individuals without a second or third vaccination were also part of the analysis. The percentage of vaccine types was calculated based on the total number of patients included in the study.

Supplementary table 6. Risk of acute transvers myelitis following COVID-19 vaccination during the observation periods of within 90 days, and 180 days in self-controlled case series analysis

| **Observation period**  **Risk Period** | **90 days** | | **180 days** | |
| --- | --- | --- | --- | --- |
|  | **Number of cases (IR)** | **IRR (95% CI)** | **Number of cases (IR)** | **IRR (95% CI)** |
| **1–42 days after each dose** |  |  |  |  |
| Risk period | 65 (5.27) | 2.22 (1.28–3.85) | 81 (2.93) | 1.98 (1.41–2.78) |
| Baseline period | 16 (2.37) | Ref | 57 (1.48) | Ref |
| **1–28 days after each dose** |  |  |  |  |
| Risk period | 48 (5.45) | 1.73 (1.11–2.71) | 58 (3.02) | 1.78 (1.27–2.50) |
| Baseline period | 32 (3.14) | Ref | 79 (1.69) | Ref |
| **1–21 days after each dose** |  |  |  |  |
| Risk period | 35 (5.15) | 1.39 (0.91–2.14) | 43 (2.94) | 1.60 (1.12–2.30) |
| Baseline period | 45 (3.70) | Ref | 94 (1.84) | Ref |

Abbreviation: IR, Incidence rate; IRR, Incidence rate ratio; CI, Confidence interval

Supplementary table 7. Subgroup analysis for the risk period of 1–21 days of acute transvers myelitis patients following COVID-19 vaccination in self-controlled case series analysis (observation period: 90 days following the first dose)

| **Subgroup** | **No. of cases** | | **IR per  person-year** | | **IRR (95% CI)** |
| --- | --- | --- | --- | --- | --- |
|  | **Risk  period** | **Baseline period** | **Risk  period** | **Baseline period** |  |
| **Age (years)** | | | | | |
| 18-29 | 6 | 4 | 6.09 | 3.36 | 1.81 (0.56–5.88) |
| 30-49 | 10 | 14 | 4.76 | 3.92 | 1.21 (0.53–2.78) |
| 50-64 | 7 | 10 | 4.76 | 3.44 | 1.56 (0.63–3.84) |
| 65-74 | 7 | 11 | 5.36 | 3.78 | 1.36 (0.54–3.44) |
| ≥75 | 5 | 6 | 4.57 | 1.59 | 1.26 (0.39–4.10) |
| **Gender** | |  |  |  |  |
| Male | 18 | 20 | 5.77 | 3.43 | 1.68 (0.91–3.12) |
| Female | 17 | 25 | 4.62 | 3.95 | 1.17 (0.64–2.14) |
| **Vaccine product immediately preceding to initial acute transverse myelitis** | | | | | |
| BNT162b2 | 17 | 18 | 4.63 | 2.36 | 1.18 (0.57–2.42) |
| ChAdOx1 | 10 | 22 | 4.73 | 3.67 | 1.29 (0.65–2.54) |
| mRNA-1273 | 6 | 3 | 6.85 | 2.43 | 2.82 (0.72–10.99) |
| Ad26.COV2.S | 1 | 2 | 6.09 | 3.58 | 1.70 (0.15–18.75) |
| NVX-CoV2373 | 0 | 0 | N/A | N/A | N/A |
| **Charlson comorbidity index score** | |  |  |  |  |
| 0–5 | 9 | 5 | 6.52 | 2.61 | 4.32 (1.61–11.60) |
| + 5 | 26 | 40 | 4.80 | 3.90 | 1.23 (0.75–2.01) |
| Abbreviation: IR, Incidence rate; IRR, Incidence rate ratio; CI, Confidence interval | | | | | |

Supplementary table 8. Subgroup analysis for the risk period of 1–28 days of acute transvers myelitis patients following COVID-19 vaccination in self-controlled case series analysis (observation period: 90 days following the first dose)

| **Subgroup** | **No. of cases** | | **IR per  person-year** | | **IRR (95% CI)** |
| --- | --- | --- | --- | --- | --- |
|  | **Risk  period** | **Baseline period** | **Risk  period** | **Baseline period** |  |
| **Age (years)** | | | | | |
| 18-29 | 7 | 3 | 5.45 | 3.34 | 1.63 (0.49–5.42) |
| 30-49 | 12 | 12 | 4.06 | 4.38 | 1.08 (0.48–2.45) |
| 50-64 | 11 | 6 | 6.30 | 2.43 | 2.59 (0.98–6.85) |
| 65-74 | 11 | 7 | 6.24 | 2.78 | 2.24 (0.88–5.69) |
| ≥75 | 7 | 4 | 5.49 | 2.97 | 1.85 (0.51–6.70) |
| **Gender** | |  |  |  |  |
| Male | 24 | 14 | 5.91 | 2.85 | 2.07 (1.05–4.08) |
| Female | 24 | 18 | 3.41 | 5.06 | 1.48 (0.82–2.68) |
| **Vaccine product immediately preceding to initial acute transverse myelitis** | | | | | |
| BNT162b2 | 20 | 15 | 4.35 | 3.84 | 1.13 (0.58–2.23) |
| ChAdOx1 | 19 | 13 | 6.93 | 2.69 | 2.58 (1.32–5.01) |
| mRNA-1273 | 7 | 2 | 4.35 | 2.11 | 2.80 (0.51–15.48) |
| Ad26.COV2.S | 1 | 2 | 4.51 | 3.99 | 1.13 (0.10–12.46) |
| NVX-CoV2373 | 0 | 0 | N/A | N/A | N/A |
| **Charlson comorbidity index score** | |  |  |  |  |
| 0–5 | 9 | 5 | 3.28 | 5.05 | 1.54 (0.57–4.16) |
| + 5 | 39 | 27 | 3.12 | 5.55 | 1.78 (1.08–2.93) |
| Abbreviation: IR, Incidence rate; IRR, Incidence rate ratio; CI, Confidence interval | | | | | |

Supplementary table 9. Subgroup analysis for the risk period of 1–21 days of acute transvers myelitis patients following COVID-19 vaccination in self-controlled case series analysis (observation period: 180 days following the first dose)

| **Subgroup** | **No. of cases** | | **IR per  person-year** | | **IRR (95% CI)** |
| --- | --- | --- | --- | --- | --- |
|  | **Risk  period** | **Baseline period** | **Risk  period** | **Baseline period** |  |
| **Age (years)** | | | | | |
| 18-29 | 6 | 10 | 3.53 | 1.80 | 1.97 (0.76–5.11) |
| 30-49 | 12 | 28 | 2.85 | 1.87 | 1.52 (0.77–3.03) |
| 50-64 | 12 | 24 | 2.90 | 1.74 | 1.66 (0.85–3.27) |
| 65-74 | 8 | 18 | 1.80 | 3.21 | 1.79 (0.78–4.10) |
| ≥75 | 5 | 14 | 2.04 | 2.40 | 1.18 (0.41–3.38) |
| **Gender** | |  |  |  |  |
| Male | 23 | 46 | 3.24 | 1.80 | 1.80 (1.10–2.95) |
| Female | 20 | 48 | 2.66 | 1.87 | 1.42 (0.84–2.40) |
| **Vaccine product immediately preceding to initial acute transverse myelitis** | | | | | |
| BNT162b2 | 22 | 51 | 2.59 | 1.90 | 1.37 (0.82–2.27) |
| ChAdOx1 | 11 | 30 | 3.06 | 1.89 | 1.61 (0.81–3.22) |
| mRNA-1273 | 9 | 10 | 2.59 | 1.48 | 2.63 (1.12–6.14) |
| Ad26.COV2.S | 1 | 3 | 4.57 | 1.73 | 2.63 (0.27–25.32) |
| NVX-CoV2373 | 0 | 0 | N/A | N/A | N/A |
| **Charlson comorbidity index score** | |  |  |  |  |
| 0–5 | 10 | 14 | 3.63 | 1.60 | 2.27 (1.03–5.01) |
| + 5 | 33 | 80 | 2.78 | 1.88 | 1.48 (0.98–2.22) |
| Abbreviation: IR, Incidence rate; IRR, Incidence rate ratio; CI, Confidence interval | | | | | |

Supplementary table 10. Subgroup analysis for the risk period of 1–28 days of acute transvers myelitis patients following COVID-19 vaccination in self-controlled case series analysis (observation period: 180 days following the first dose)

| **Subgroup** | **No. of cases** | | **IR per  person-year** | | **IRR (95% CI)** |
| --- | --- | --- | --- | --- | --- |
|  | **Risk  period** | **Baseline period** | **Risk  period** | **Baseline period** |  |
| **Age (years)** | | | | | |
| 18-29 | 7 | 9 | 3.12 | 1.79 | 1.75 (0.69–4.44) |
| 30-49 | 14 | 26 | 1.90 | 2.51 | 1.32 (0.69–2.51) |
| 50-64 | 16 | 20 | 2.89 | 1.62 | 1.79 (0.93–3.44) |
| 65-74 | 13 | 13 | 3.90 | 1.42 | 2.75 (1.26–6.03) |
| ≥75 | 11 | 8 | 3.16 | 1.71 | 1.85 (0.74–4.61) |
| **Gender** | |  |  |  |  |
| Male | 30 | 39 | 3.22 | 1.67 | 1.93 (1.19–3.13) |
| Female | 28 | 40 | 2.83 | 1.72 | 1.65 (1.02–2.65) |
| **Vaccine product immediately preceding to initial acute transverse myelitis** | | | | | |
| BNT162b2 | 26 | 47 | 2.37 | 1.92 | 1.23 (0.76–1.99) |
| ChAdOx1 | 21 | 20 | 4.33 | 1.37 | 3.17 (1.75–5.73) |
| mRNA-1273 | 10 | 9 | 3.21 | 1.51 | 2.13 (0.88–5.18) |
| Ad26.COV2.S | 1 | 3 | 3.38 | 1.81 | 1.86 (0.19–17.92) |
| NVX-CoV2373 | 0 | 0 | N/A | N/A | N/A |
| **Charlson comorbidity index score** | |  |  |  |  |
| 0–5 | 10 | 14 | 2.74 | 1.78 | 1.55 (0.70–3.40) |
| + 5 | 48 | 65 | 3.08 | 1.68 | 1.84 (1.26–2.67) |
| Abbreviation: IR, Incidence rate; IRR, Incidence rate ratio; CI, Confidence interval | | | | | |
